# Role of MALAT1 as a Prognostic Factor for Survival in Various Cancers: A Systematic Review of the Literature with Meta-Analysis

**DOI:** 10.1155/2015/164635

**Published:** 2015-09-02

**Authors:** Yao Wei, Ben Niu

**Affiliations:** ^1^Department of Oncology, Tongji Hospital, Tongji Medical College, Huazhong University of Science and Technology, Wuhan 430030, China; ^2^Department of Anesthesiology and Pain Medicine, Tongji Hospital, Tongji Medical College, Huazhong University of Science and Technology, Wuhan 430030, China

## Abstract

*Objectives*. The expression of metastasis-associated lung adenocarcinoma transcript 1 (MALAT1), a highly abundant and ubiquitously expressed long noncoding RNA (lncRNA), influences clinical parameters and may have prognostic value in cancer. This meta-analysis evaluated the prognostic role of MALAT1 in various cancers. *Materials and Methods*. Systematic literature searches of PubMed and EMBASE databases were conducted for eligible studies of the prognostic role of MALAT1 in cancer. Overall survival (OS), disease-specific survival (DSS), and disease-free survival (DFS) were analyzed. Summary hazard ratios (HRs) and 95% confidence intervals (95% CIs) were assessed to evaluate the influence of MALAT1 expression on patient prognosis. *Results*. Nine studies with a total of 932 patients were included in the analysis. Elevated MALAT1 expression was significantly correlated with poor OS (HR 2.02; 95% CI: 1.62–2.52; *P* < 0.001; *I* ^2^ = 0%). Subgroup analysis indicated that tumor type, histology type, ethnicity, and measurement technique did not affect the prognostic value of MALAT1 for OS. The HR of elevated MALAT1 for DFS was 2.78 (95% CI: 1.87–4.15; *P* < 0.001; *I* ^2^ = 0%). *Conclusions*. Elevated MALAT1 expression is correlated with poor OS in various types of cancer, suggesting that this gene is a prognostic factor for different types of cancer.

## 1. Introduction

Noncoding RNAs (ncRNAs) are RNAs that do not encode proteins and play important roles [1, 2]. Small ncRNAs, such as microRNAs (miRNAs), have been extensively studied in association with many human diseases [3]. However, long noncoding RNAs (lncRNAs), which are commonly defined as RNA molecules with lengths of greater than 200 nucleotides, have a broad range of molecular and cellular functions via distinct mechanisms that are not yet fully understood [4]. Accumulating reports of aberrant lncRNA expression in various cancers indicate that lncRNAs may substantially contribute to cancer development [5].

Metastasis-associated lung adenocarcinoma transcript 1 (MALAT1), also named nuclear-enriched abundant transcript 2 (NEAT2), is a widely expressed lncRNA that is greater than 8000 nucleotides in length. MALAT1 was first identified as a factor indicating high metastatic potential and poor prognosis in a study of gene expression differences in stage I non-small-cell lung cancer (NSCLC) with/without metastasis [6]. MALAT1 has since been associated with several human neoplasms, including lung [7, 8], liver [9], renal [10], colorectal [11], gastric [12], breast [13], cervical [14], pancreatic [15], and bladder cancers [16], uterine endometrial stromal sarcoma [17], glioma [18], and osteosarcoma [19]. The expression of this gene may affect the clinical parameters and prognosis of cancer patients. However, most studies assessing the implications of MALAT1 expression in cancer have been limited by small sample sizes and have produced controversial results. Therefore, we performed a systematic review and quantitative meta-analysis to assess the prognostic role of MALAT1 expression in various cancers.

## 2. Materials and Methods

### 2.1. Literature Search Strategy

Electronic searches of PubMed and EMBASE were performed using the following keywords: “MALAT1,” “MALAT-1,” “MALAT1 long non-coding RNA, human,” “metastasis-associated lung adenocarcinoma transcript 1,” “NEAT2,” “NEAT2 long non-coding RNA, human,” “carcinoma,” “neoplasm,” “cancer,” “prognosis,” “prognostic,” and “outcome,” without any limits. The reference lists of the retrieved articles were searched manually. The search ended in February 2015.

### 2.2. Eligibility Criteria

The eligibility criteria for the studies were as follows: (1) evaluation of a link between MALAT1 expression and prognosis of patients with any type of cancer; (2) reporting of outcomes, including overall survival (OS), disease-specific survival (DSS), or disease-free survival (DFS); (3) reporting of hazard ratios (HRs) and 95% confidence intervals (CIs) or data that could be used to calculate these values; and (4) full papers in English. Nonhuman research, duplicated studies, reviews, letters, comments, and single case reports were omitted.

### 2.3. Data Extraction

Yao Wei and Ben Niu reviewed each eligible study and extracted the data. The following information was collected: author; year of publication; country; cancer type and stage; number of patients; techniques used to assess MALAT1 expression; follow-up period; and cut-off values, HRs, and corresponding 95% CIs for OS, DSS, or DFS. HRs were directly determined by multivariate analysis in some studies, whereas others provided Kaplan-Meier survival curves. For the latter studies, we first extracted several specific points from the survival curves using Engauge Digitizer version 4.1 to obtain two lists of survival rates at specific time points from the two survival curves. We then input the extracted survival rates at specific time points into the spreadsheet developed by Tierney et al. to calculate the HR and 95% CI [20]. Finally, we produced an approximated curve and compared it with the original curves to confirm the accuracy of our data extraction.

### 2.4. Quality Assessment

Yao Wei and Ben Niu performed a quality assessment of the included studies according to the guidelines of Hayden et al. [21]. This assessment included evaluations of the following six items: study participation, study attrition, prognostic factor measurements, confounding measurements and relevant adjustments, outcome measurements, and analysis. The results for each item were described as “yes,” “no,” “partly,” or “unsure.” Consensus was achieved after each item was discussed, and the overall risk was determined for each potential bias.

### 2.5. Statistical Analysis

We evaluated the impact of MALAT1 expression on clinical prognosis by examining the HRs and corresponding 95% CIs. An observed HR of >1 indicated poorer prognosis in patients with elevated MALAT1 expression. The results were considered statistically significant when the 95% CI did not overlap with 1. We used *I* ^2^ statistic to assess statistical heterogeneity between studies. Significant heterogeneity was defined as *I* ^2^ > 50%. If there was no significant heterogeneity between studies, the fixed-effects model was used. Otherwise, the random-effects model was used. Subgroup analysis with stratification by tumor type, histology type, ethnicity, and measurement method was conducted. Sensitivity analysis was performed with sequential omission of each study. Probable publication bias was estimated by Begg's test and by constructing a funnel plot. All *P* values were two tailed, and *P* < 0.05 was considered statistically significant. Review Manager version 5.3 and STATA software version 11.0 (Stata Corporation, College Station, Texas, USA) were used to conduct statistical analysis.

## 3. Results

### 3.1. Included Studies and Characteristics

**Figure 1:**
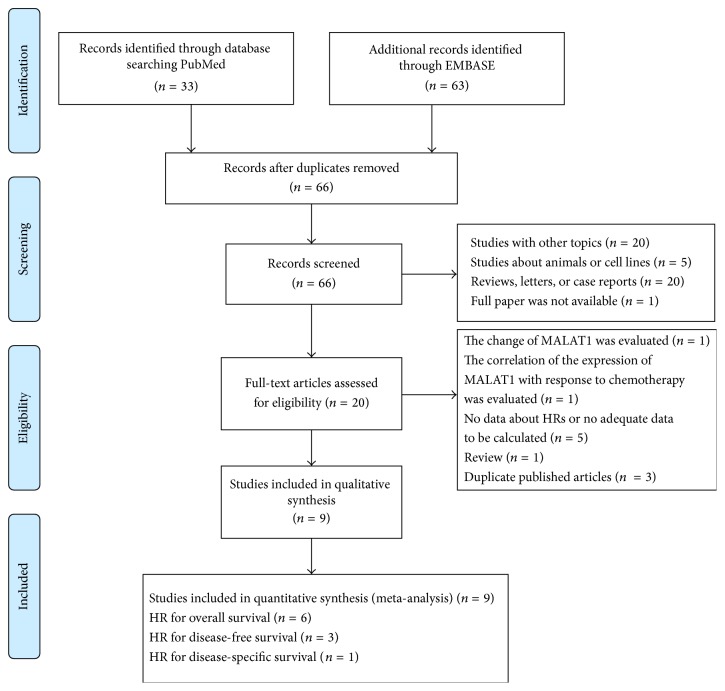
Flow diagram of the meta-analysis.

A flow diagram of the literature search process is presented in Figure 1. Sixty-six papers were obtained by PubMed and EMBASE searches. Forty-six articles were excluded after the abstracts were reviewed. Additional 11 articles were excluded after the full papers were assessed. Ultimately, 9 articles were included in this meta-analysis.

The clinical characteristics of the 9 included studies are summarized in Table 1. The articles were published between 2011 and 2015 with sample sizes ranging from 45 to 150 and included a total of 932 participants. Six of the studies enrolled more than 100 participants each. The participants in the studies were from China, Germany, and Japan. Seven different types of cancer were examined (2 studies of NSCLC, 2 of pancreatic cancer, 1 of clear cell renal cell carcinoma, 1 of gastric cancer, 1 of colorectal cancer, 1 of hepatocellular carcinoma, and 1 of glioma). HRs and 95% CIs were directly retrieved from 8 studies and were calculated from survival curves for 1 study.

The results of the quality assessment are presented in Table 2. The key baseline characteristics of the study sample were not adequately described in Shen LQ's study. Further, the key characteristics of participants lost to follow-up were not described in Schmidt LH's study. In addition, Lai MC's study did not include a well-defined cut-off MALAT1 expression level. The duration of follow-up was not clearly described in 3 studies (those of Zhang HM, Schmidt LH, and Shen LQ). None of the studies described important confounders, such as subsequent treatments.

### 3.2. Primary Outcome: OS

**Figure 2:**
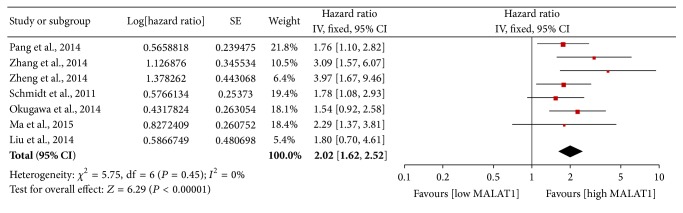
Forest plots of the HRs of elevated MALAT1 expression for overall survival for the included studies. Log⁡[hazard  ratio]: logarithm of the hazard ratio; SE: standard error; weight: the weight given to each study by the inverse of the variance of the hazard ratio. IV: inverse variance; fixed: fixed-effects analysis.

**Figure 3:**
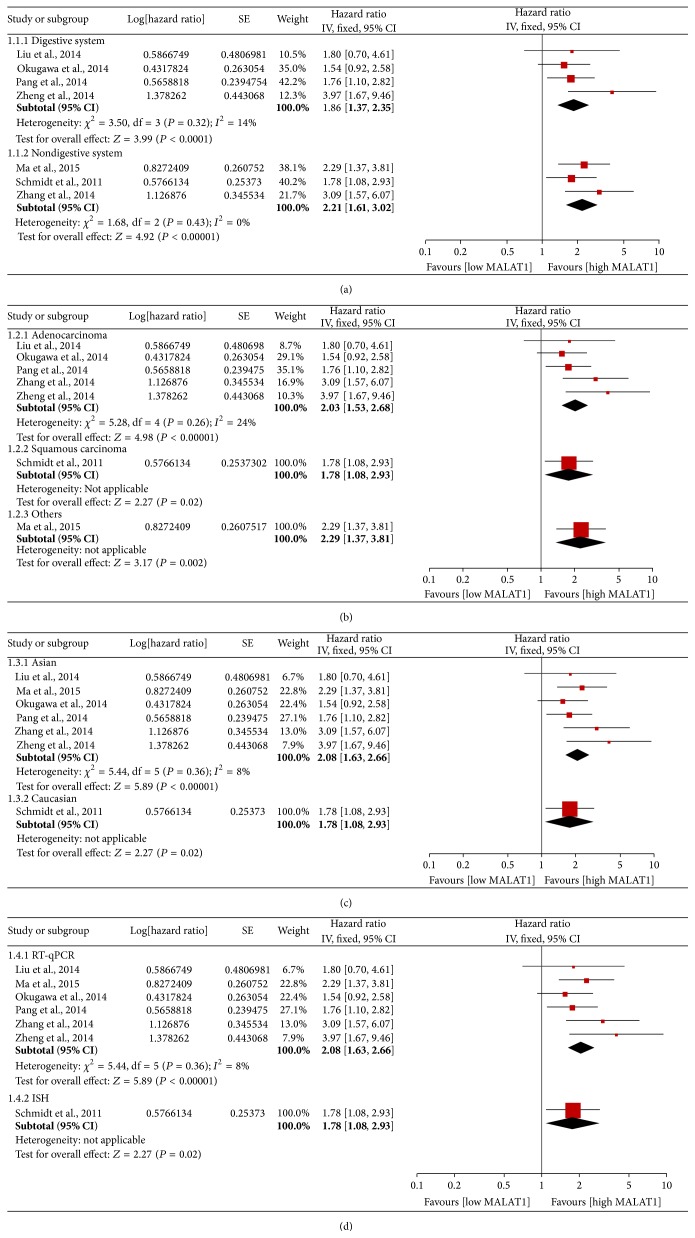
Forest plots of the HRs of elevated MALAT1 expression for overall survival in different subgroups. (a) Subgroup analysis of HRs for overall survival by tumor type. (b) Subgroup analysis of HRs for overall survival by histology type. (c) Subgroup analysis of HRs for overall survival by region. (d) Subgroup analysis of HRs for overall survival by measurement method. Log⁡[Hazard  Ratio]: logarithm of hazard ratio; SE: standard error; weight: the weight given to each study by the inverse of the variance of the hazard ratio. IV: inverse variance; fixed: fixed-effects analysis.

The main results of the meta-analysis are presented in Table 3. Seven studies including 793 participants reported HRs for OS or DSS. HRs and 95% CIs were directly determined by multivariate analysis in all 7 studies. Elevated MALAT1 expression was predictive of poor OS (HR 2.02; 95% CI: 1.62–2.52; *P* < 0.001; Figure 2). The fixed-effects model was used because of evidence of nonsignificant heterogeneity (*P* = 0.452, *I* ^2^ = 0%) among the studies. The effects of elevated MALAT1 expression on OS among different tumor types, histology types, and ethnicities and according to different measurement methods are presented in Table 3 and Figure 3.

**Figure 4:**
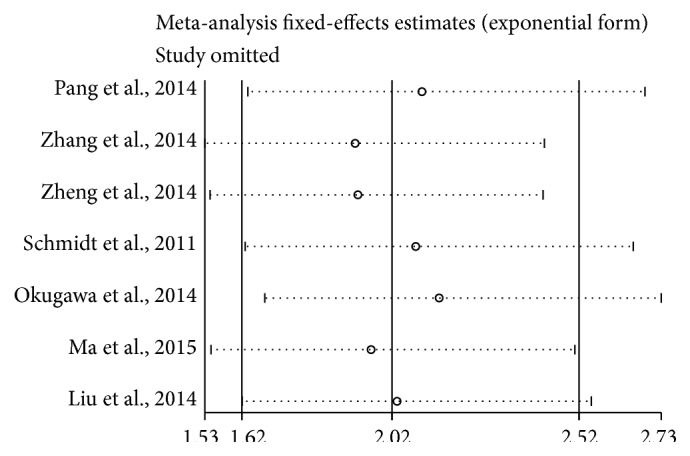
Sensitivity analysis of the pooled HRs of MALAT1 expression for overall survival for the included studies.

Sensitivity analysis indicated that the pooled HR was not significantly affected by the exclusion of any of the studies (Figure 4).

**Figure 5:**
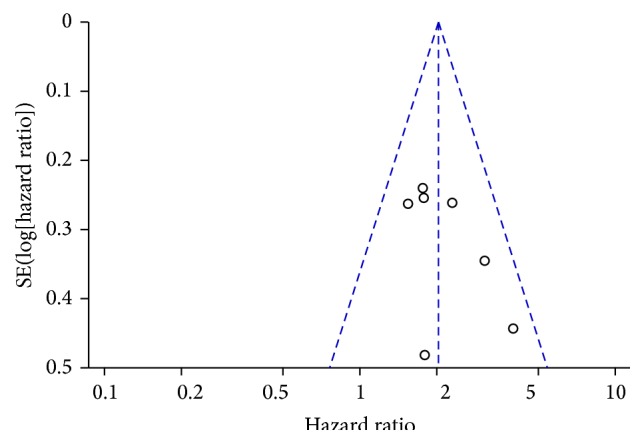
Funnel plot for the publication bias test of the included studies for MALAT1 expression and overall survival. SE(log⁡[hazard  ratio]): standard error of the logarithm of the hazard ratio. Each dot represents a study. The vertical line represents the summary estimate of the hazard ratio. The diagonal lines represent the 95% confidence limits around the summary hazard ratio.

The funnel plot indicated no significant asymmetry (Figure 5). *P* value of Egger's regression intercepts was 0.170. Therefore, no significant publication bias was detected in this meta-analysis.

### 3.3. Secondary Outcome: DFS

**Figure 6:**
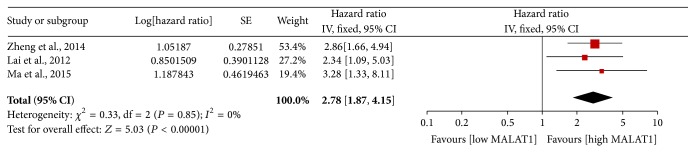
Forest plots of the HRs of elevated MALAT1 expression for disease-free survival for the included studies. Log⁡[hazard  ratio]: logarithm of the hazard ratio; SE: standard error; weight: the weight given to each study by the inverse of the variance of the hazard ratio. IV: inverse variance; fixed: fixed-effects analysis.

Three studies including 285 participants reported HRs for DFS (Table 3). The HRs and 95% CIs were determined by multivariate analysis in 2 studies and calculated from the survival curve in 1 study. Elevated MALAT1 expression was predictive of decreased DFS (HR 2.78; 95% CI: 1.87–4.15; *P* < 0.001; Figure 6). The fixed-effects model was used because of evidence of nonsignificant heterogeneity (*P* = 0.848, *I* ^2^ = 0.0%) among the studies.

## 4. Discussion

The prognostic role of MALAT1 in cancer was evaluated by a meta-analysis of 9 studies including 932 participants. Elevated MALAT1 expression was indicative of poor prognosis in patients with various types of cancer. The pooled HR for OS was 2.02 (95% CI: 1.62–2.52; *P* < 0.001), and the HRs were similar among the different tumor types, histology types, races, and measurement methods. There was evidence of nonsignificant heterogeneity (*P* = 0.452, *I* ^2^ = 0%) among the studies. Sensitivity analysis demonstrated that the pooled HR was not significantly affected by the exclusion of any of the studies. No publication bias was detected. Elevated MALAT1 expression was associated with poorer DFS, although the role of MALAT1 in cancer development requires further evaluation.

The mechanism underlying the relationship between elevated MALAT1 expression and poor prognosis in patients with various types of cancer is uncertain. MALAT1 may regulate alternative splicing. MALAT1 interacts with serine/arginine proteins and influences the distribution of splicing factors in nuclear speckle domains [22]. In MALAT1-depleted cells, the expression of an oncogenic transcription factor, B-MYB (Mybl2), which is involved in G2/M progression, is reduced because of the aberrant binding of splicing factors and abnormal alternative splicing [23]. However, its capacity for regulating alternative splicing could not be confirmed in MALAT1 knockout mice or lung cancer cells [24, 25]. MALAT1 has critical and specific functions in regulating the expression of several target genes. Depletion of MALAT1 from HeLa cells represses the expression of several genes, including 2′-5′-oligoadenylate synthetase-like protein, interferon-induced protein 44, and serine peptidase inhibitor Kazal type 4 [26]. In lung cancer, MALAT1 regulates a series of metastasis-associated genes, and thus the migratory ability of MALAT1-deficient cells is impaired [24]. MALAT1 is also involved in the transcriptional control of cell cycle gene expression and is required for the recruitment of coactivators by polycomb 2 (Pc2) to the promoters of cell cycle control genes [27]. Decreases in the levels of epithelial-mesenchymal transition- (EMT-) associated ZEB1, ZEB2, and Slug and an increase in that of E-cadherin occur upon downregulation of MALAT1 [16].

The elucidation of prognostic factors is crucial for the identification of high-risk patients who are good candidates for individual therapy. The results of our meta-analysis indicate that elevated MALAT1 expression affects the prognosis of cancer patients, and these findings should promote the development of adequately designed prospective studies. Furthermore, this gene might represent a potential therapeutic target. MALAT1 knockdown strategies may be developed for antimetastatic therapy. Studies of the function of MALAT1 in the vasculature have revealed that its inhibition induces a switch from an endothelial cell phenotype to a promigratory but antiproliferative state, resulting in impaired endothelial cell proliferation* in vitro* and* in vivo* and reduced retinal vessel growth [28]. MALAT1 inhibition may elicit an antiangiogenic effect in the hypoxic tumor environment.

There are several limitations of our study. First, the pooled survival data were calculated based on results reported for patients with various types of cancer because the available studies were heterogeneous. The prognostic role of MALAT1 in each type of cancer could not be evaluated because of the limited data available. Second, there was a bias towards Asian patients because 7 of 9 studies were from China and one study was from Japan. Third, the techniques used to identify MALAT1 expression could have led to possible bias. In most of the studies, MALAT1 expression was detected by real-time quantitative PCR, except in Schmidt LH's study, which employed* in situ* hybridization. However,* in situ* hybridization is far less sensitive and quantitative than real-time quantitative PCR. Moreover, cut-off values were not reported in some studies, and those that were reported were inconsistent among studies, which may have reduced the power for detecting a real association. Fourth, most of the included studies reported significant results because studies with nonsignificant results may not be published. Moreover, some authors described significant results of subgroup analyses but did not report the results for other nonsignificant subgroups. Fifth, subsequent treatment after surgery differed among the studies and can greatly influence survival, leading to some heterogeneity. Sixth, OS was the endpoint in a majority of the studies, except for Liu JH's study, which used DSS as the endpoint. This study was also included in analysis of the pooled HR for OS, which may not have been reasonable. OS only represents an approximation of DSS if the proportion of cancer-unrelated deaths is small. However, the pooled HR was not significantly affected by the removal of Liu JH's study in the sensitivity analysis.

In conclusion, MALAT1 may be a prognostic factor for patients with various types of cancer. Further studies are needed to confirm its precise role among other known prognostic factors for specific types of cancer.

## Figures and Tables

**Table 1:** Characteristics of the studies included in the meta-analysis.

First author	Year	Country	Cancer type	Stage	Sample size (*n*)	Method	Cut-off	Follow-up (months)	Outcome	Survival analysis
Pang [15]	2015	China	Pancreatic cancer	I–IV	126	RT-qPCR	Median value of 6.23	5–60	OS	Univariate and multivariate
Zhang [10]	2014	China	Renal cell carcinoma (clear cell)	I–IV	106	RT-qPCR	Mean value of 3.85	NA	OS	Univariate and multivariate
Liu [29]	2014	China	Pancreatic duct adenocarcinoma	I–IV	45	RT-qPCR	Mean value (NA)	24–36	DSS	Univariate and multivariate
Zheng [11]	2014	China	Colorectal cancer	II-III	146	RT-qPCR	6.15 (MALAT1/GAPDH ratio)	11–72.8	DFS, OS	Univariate and multivariate
Schmidt [7]	2011	Germany	Non-small-cell lung cancer (squamous cell)	I–III	102	ISH	A large gene copy cluster in 50% of cells	NA	OS	Univariate and multivariate
Okugawa [12]	2014	Japan	Gastric cancer	I–IV	150	RT-qPCR	Threshold of 0.985	1–78	OS	Univariate and multivariate
Shen [8]	2014	China	Non-small-cell lung cancer	NA	79	RT-qPCR	Mean value (NA)	NA	DFS	Univariate
Lai [9]	2012	China	Hepatocellular carcinoma	NA	60	RT-qPCR	NA	18.6 (median)	DFS	Univariate and multivariate
Ma [18]	2015	China	Glioma	I–IV	118	RT-qPCR	Median value of 5.18	5 years	OS	Univariate and multivariate

RT-qPCR: real-time quantitative PCR; ISH: *in situ* hybridization; OS: overall survival; DFS: disease-free survival; DSS: disease-specific survival; NA: not available.

**Table 2:** Quality assessment of the studies included in the meta-analysis.

First author	Study participation	Study attrition	Prognostic factor measurement	Outcome measurement	Confounding measurements and adjustments	Analysis
Pang [15]	Yes	Yes	Yes	Yes	Partly	Yes
Zhang [10]	Yes	Yes	Yes	Partly	Partly	Yes
Liu [29]	Yes	Yes	Yes	Yes	Partly	Yes
Zheng [11]	Yes	Yes	Yes	Yes	Partly	Yes
Schmidt [7]	Yes	Partly	Yes	Partly	Partly	Yes
Okugawa [12]	Yes	Yes	Partly	Yes	Partly	Yes
Shen [8]	Partly	Yes	Yes	Partly	Partly	Yes
Lai [9]	Yes	Yes	Partly	Yes	Partly	Yes
Ma [18]	Yes	Yes	Yes	Yes	Partly	Yes

**Table 3:** The main results of the pooled analyses.

Survival	Variables	Number of studies	Number of patients	HR	95% CI	*P* value	Heterogeneity (*I* ^2^, %)
OS	All	**7**	**793**	**2.02**	**1.62–2.52**	**<0.001**	**0**
	Tumor type						
	Digestive system	4	467	1.86	1.37–2.53	<0.001	14.2
	Nondigestive system	3	326	2.21	1.61–3.02	<0.001	0
	Histology type						
	Adenocarcinoma	5	573	2.03	1.53–2.68	<0.001	24.2
	Squamous carcinoma	1	102	1.78	1.08–2.92		
	Others	1	118	2.29	1.37–3.81		
	Ethnicity						
	Asian	6	691	2.08	1.63–2.66	<0.001	8.1
	Caucasian	1	102	1.78	1.08–2.92		
	Method						
	RT-qPCR	6	691	2.08	1.63–2.66	<0.001	8.1
	ISH	1	102	1.78	1.08–2.92		
DFS	All	3	285	2.78	1.87–4.15	<0.001	0

RT-qPCR: real-time quantitative PCR; ISH: *in situ* hybridization; OS: overall survival; DFS: disease-free survival.
